# IMMUNE RESPONSE UPON THE ADMINISTRATION OF RECOMBINANT PROTEIN ANTIBODIES Ag-38 KDa *Mycobacterium tuberculosis* AND RIFAMPICIN *EX-VIVO*

**DOI:** 10.21010/Ajid.v16i2.8

**Published:** 2022-05-06

**Authors:** Tri Yudani Mardining Raras, Almira Fahrinda, Yuliati, Dwi Yuni Nurhidayati, Hidayat Sujuti, Sumarno Reto Prawiro

**Affiliations:** 1 Department of Biochemistry and Molecular Biology, Faculty of Medicine, Universitas Brawijaya Malang. Indonesia; 2 Master Program in Biomedical Science, Faculty of Medicine, Universitas Brawijaya. Malang Indonesia; 3 Department of Clinical Microbiology, Faculty of Medicine, Universitas Brawijaya. Malang Indonesia

**Keywords:** Antibody, Lymphocyte, Macrophage, *Mycobacterium tuberculosis*, Recombinant, Rifampicin

## Abstract

**Background:**

Development a granuloma model resembling latent tuberculosis *in vitro* is needed with a fast and efficient time to be used as an effective therapy. This study aimed to form efficient granulomas, increase cellular immunity and humoral immunity, and evaluate growth on media using recombinant protein antibody Ag38kDa, Rifampicin, and a combination of both. Peripheral Blood Mononuclear Cell (PBMC) *in vitro* is derived from a healthy individual separated from monocytes and lymphocytes.

**Materials and methods:**

Monocytes are matured into macrophages and then combined macrophages and lymphocytes to the Roswell Park Memorial Institute (RPMI) medium. Flow cytometry analysis was used to count the number of cells, and cytokine levels were measured using ELISA. The result from the treatment was planted on the Lowenstein-Jensen medium.

**Results:**

Granulomas-like aggregates was formed after one-day post-infection with *Mycobacterium tuberculosis* (*M.tb*). A significant increase in immune response occurred in the number of macrophages, Th1, and Tregs in the combination group compared to the *Mtb* infection group. The number of Th2 and Th17 cells in the combination group was compared with the control but not significantly. TNF-α cytokine levels increased in the combination group compared to *Mtb* infection, while in IL-4, we found between all groups, there was no significant difference. Bacterial colonies on culture in the Lowenstein-Jensen medium were only seen in positive controls.

**Conclusion:**

Our study concluded that administration of a combination between Ag38kDa recombinant antibody and rifampicin could inhibit granuloma formation and enhance immune response.

## Introduction

Tuberculosis (TB) is an infectious disease included in the top 10 causes worldwide, even being the leading cause of death in the single infectious agent category, above HIV/AIDS (WHO, 2020). Furthermore, tuberculosis is a significant threat to the world’s population because a quarter of the world’s population has been latently infected with *Mycobacterium tuberculosis* (Cohen *et al.*, 2019).

*Mycobacterium tuberculosis* enters the respiratory tract and activates dendritic cells and macrophages. The activation of these Antigen Presenting Cell (APC) cells causes proinflammatory cytokines such as TNF to produce cytokines and chemokines. *Mtb* infection results in granulomas in the lungs (Lin *et al.*, 2013). Granulomas formation due to T cell responses such as cytotoxic and NK cells can kill infected cells. Th1 cells produce a combination of IL-2, interferon-γ, and TNF and Th17 cells that produce IL-17, and regulatory T cells (Treg) that produce IL-17. In addition, it can produce IL-10 or TGF-β and inhibit the proliferation and production of cytokines by other cells (Flynn, Chan, and Lin, 2011). Th2 cells secrete cytokines such as interleukin 4 (IL-4), which play a role in downregulating the protective response to Th1 and increasing in advanced stages of tuberculosis (Vidyarani *et al.*, 2006).

One of the antibiotics currently used in treating *Mtb* is Rifampicin (Palomino and Martin, 2014). Rifampicin can penetrate granulomas to kill *Mtb* and sterilize *Mtb* bacteria (Lohrasbi *et al.*, 2018). Rifampicin inhibits RNA DNA-dependent polymerase in bacteria (Calvori, Frontali, Leoni, and Tecce, 1965). Treatment using antibiotics takes a relatively long time, so low treatment adherence (Hoagland, Liu, Lee and Lee, 2016).

Ag38 kDa protein contains two *Mtb*-specific B cell epitopes, the most potent immunogens because they have high specificity and sensitivity (Wilkinson, Haslov, and Rappuoli, 1997). Therefore, we developed Ag38kDa recombinant protein injected into mice to generate Ag38kDa recombinant protein antibodies. The use of recombinant protein antibody Ag38kDa combined with Rifampicin is expected to be an additional treatment in increasing the effectiveness of therapy and shortening the duration of TB treatment.

The Study of *in vitro* granuloma formation in PBMC was pioneered by Puissegur *et al*. Using a surface coating of sepharose takes 4-5 days after being exposed to *Mtb*. Furthermore, Kapoor (2013), modelled granulomas to resemble dormancy, and resuscitation occurred 5-8 days after infection. Our study aims to form granulomas more with a fast and efficient time by first maturating monocytes into macrophages, with exposure of the recombinant protein antibody Ag38kDa, Rifampicin, and a combination of Rifampicin with antibodies. Cellular and humoral immune responses stimulated by combination antibody Ag38kDa and Rimfapisin, namely Th1, Th2, Th17, Treg, macrophage cells, humoral immunity, namely IL-4 and TNF-a, inhibit the formation of granulomas and observe growth results on Lowenstein-Jensen (LJ) medium after treatment exposure.

## Materials and methods

### PBMC Isolation Procedure

The blood sample comes from a healthy individual with evidence of a complete blood count and chest X-ray in normal conditions. The blood was diluted using PBS, then put into a centrifugation tube and added Ficoll Hipaque d=1,077 g/mL in a ratio of 1:1, then centrifuged at 1600 rpm for 30 minutes. After that, the blood in the tube formed five layers, namely plasma, PBMC, Ficoll Hipaque, granulocytes, and erythrocytes. The PBMC layer was taken using a micropipette and put into a tube. Furthermore, the PBMC layer was washed with 10 mL of PBS, centrifuged at 1200 rpm for 10 minutes, added RBC lysis buffer, and washed with PBS 2 times. PBMCs were cultured on 24-well plates in a complete medium consisting of 1x RPMI 1640 with the addition of 2 mM l-glutamine, 10% FBS, 100 g/mL Streptomycin, and 100 u/mL Penicillin, each well filled with 1 mL, then incubated at 37°C, 5% CO2 (**Fuss, Kanof, Smith, & Zola, 2009**).

### Maturation of Monocytes to Macrophages

PBMC cells that adhere to the plate base are considered monocytes, while those not attached are deemed to contain lymphocytes and are separated in other well plates with a complete medium. The attached monocytes were washed with sterile PBS and centrifuged at 1200 rpm for 10 minutes twice, then cultured on new well plates in a complete medium. Every day the medium is replaced with a new complete medium, up to seven days. As a result, monocytes have become macrophages. Then lymphocytes in separate wells were added to the same wells with the macrophages to form a fundamental component. After that, we added extracellular matrix (ECM) solution to each well in a ratio of 1:6 to the complete medium. The ECM consisted of 1 L COL4A3, 50 L 10X DPBS, 4 L fibronectin, and 10 L 1N NaOH and added sterile PBS to 1 mL (Kapoor *et al.*, 2013). There were five treatment groups: control, *Mtb* infection, rifampin administration, Ag38kDa antibody, and rifampin combination group with Ag38kDa antibody.

### Overexpression of Ag38kDa

Recombinant protein Ag38kDa was obtained from the Biomedical Laboratory, Faculty of Medicine, Universitas Brawijaya Malang, Indonesia. The pMB38 plasmid (Raras & Lyrawati, 2011), carrying the *pab* gene, was expressed in *E. coli* strain BL21-(DE-3) carrying the Ag38kDa recombinant protein plasmid was purified using Column Nited (**Raras, Sholeh, & Lyrawati, 2014**).

### Production of Ag38kDa recombinant protein antibodies

The recombinant Ag38kDa protein was emulsified with Freud’s Complete Adjuvant (CFA), and then 100 µL was injected intraperitoneally into mice. Booster injections were carried out in the second, third-, and fourth-weeks using antigen emulsified with Incomplete Freud’s Adjuvant (IFA). A week after, booster serum was taken from the heart of mice. Blood was taken from five mice, put in a sterile tube, and centrifuged at 10,000 rpm for 5 minutes. Serum was placed in a sterile tube and stored at -20°C.

### Mycobacterium tuberculosis infection on culture media

Local strain *Mtb* from Syaiful Anwar Hospital Malang Indonesia was suspended with 10^8^ bacteria/mL with sterile PBS. *Mtb* bacteria were added to wells with a positive control treatment group with Multiplicity of Infection (MOI) 0.1, then incubated using a candle jar at 37°C. After that, they were observed every day to form granulomas for seven days.

### Administration of Rifampicin

The Rifampicin used is rifampicin tablets for the treatment of TB. Rifampicin was dissolved in DMSO with a rifampicin concentration of 5 g/ml. Rifampicin was administered on the 4th day after the granuloma was formed, then incubated for three days at a 37^o^C candle jar.

### Administration of Ag38 kDa recombinant protein antibodies

Recombinant Protein Antibody *Mtb* bacteria were coated with Ag38kDa recombinant protein antibody with a dilution ratio of 1:500, then slowly shaken for 30 minutes. The coating results were inserted into the well and incubated at 37°C in candle jars. Every day, They were observed under an inverted microscope.

### Administration of a combination of Ag38 kDa recombinant protein antibodies and Rifampicin

Recombinant protein antibody, Rifampicin, and Ag38 kDa recombinant protein antibody were coated with *Mtb* bacteria and then gently shaken for 30 minutes. The coating result were put into a plate containing macrophages and lymphocytes, and ECM on the first day. Next, gently shaken and incubated for three days at 37°C in a candle jar every day and observed using an inverted microscope. On the third day, 5 g/ml of Rifampicin was added, incubated for three days at 37°C in a candle jar, and observed using an inverted microscope.

### Flow Cytometry Analysis

Cells from all treatment groups were taken on the sixth day after treatment and centrifuged at 3000 rpm for 12 minutes. The pellets formed were used for examination using BD FACS Melody flowcytometry. The pellets were washed using PBS and centrifuged at 2500 rpm for 5 minutes. Staining using antihuman antibodies CD4+ FITC & IFN-γ PE for Th1, CD4+ FITC and IL-4 PE for Th2, CD4+ FITC and IL-17A PE for Th17, CD4+ FITC, CD25 PE/Cy5, and FoxP3 PE for Treg, and CD14 for macrophage markers (BioLegend, San Diego, CA, USA).

### ELISA Analysis

Samples were obtained from culture supernatants in all treatments. Cytokine levels were measured using the Legend MaxTM Human IL-4 ELISA kit (BioLegend, San Diego, CA, USA) and the Legend MaxTM Human TNF-α ELISA kit pre-coated plate (BioLegend, San Diego, CA, USA). The test was carried out according to the procedure indicated on the kit.

## Statistical analysis

The ANOVA test determined the difference in response in each treatment group. Data are presented in Mean±SD. Statistical analysis was done using SPSS 23 version with p < 0.05 was considered different significant.

### Ethical Approval

The Faculty Medicine of the Ethics Committee, Universitas Brawijaya Malang Indonesia, approved the study via an approval letter No 363/ EC/KEPK/10/2019.

## Results

**Figure 1:**
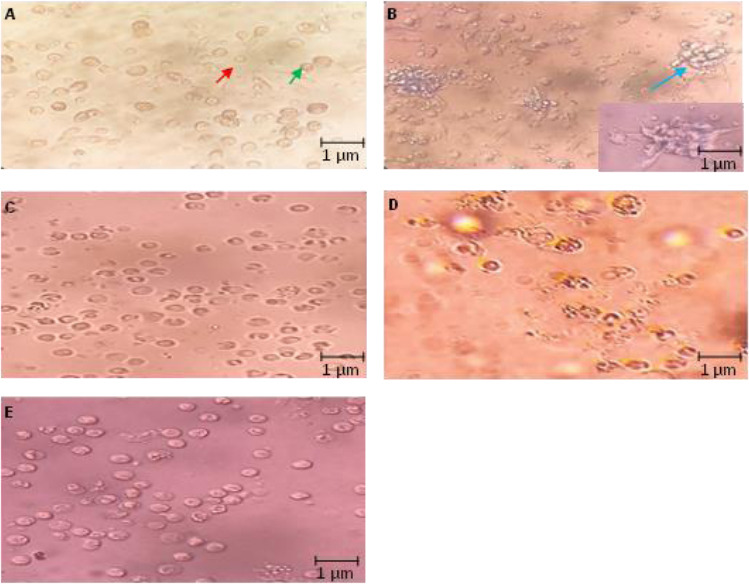
Microscopic view of granuloma formation in PBMC culture (400x magnification). A = Control; B = Treatment of *Mtb* bacterial Infection; C = Ag38kDa Recombinant Antibody Treatment; D = rifampicin treatment; E = Combination treatment of recombinant antibodies and Rifampicin. Green arrow = Macrophages, red arrow = Lymphocytes, blue arrow = Granuloma Fig 1 a and c are not visible.

To develop granuloma model, mature macrophage were combined with lymphocyte and infected with Mycobacteria. The structural and immunological changes of macrophage throughout the infection phase were analysed. Apparently the cells tend to clump to form granuloma-like aggregates since day 1 post infection. Nevertheless, only low number of cellular aggregates with small size. Microscopic examination showed the cellular structure of the granuloma formation as presented in Figure 1.

**Figure 2:**
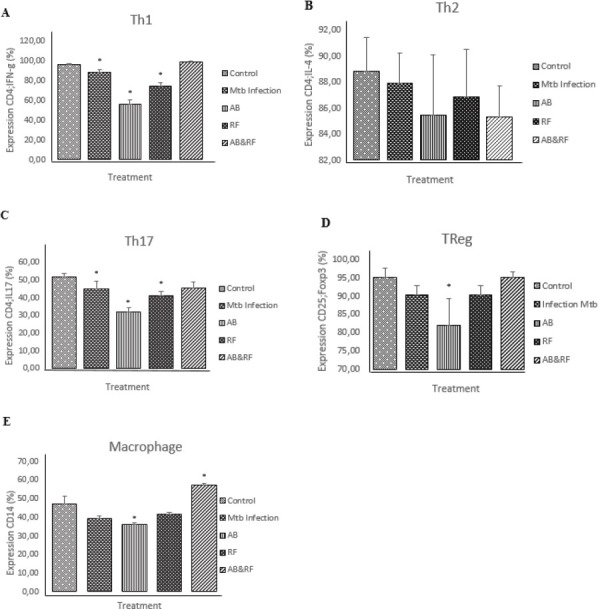
Total Cellular Immunity treated with Ag38kDa recombinant protein antibody (AB), Rifampicin (RF), and the combination of Ag38kDa recombinant protein antibody with Rifampicin (AB&RF) compared with control and *Mtb* infection in PBMC culture. We analyzed the data using one-way analysis of variance (ANOVA) and Games-howel post hoc test as validation *P<0,05 compared to control and Infection *Mtb* group.

We measured cellular immunity in each treatment, namely control treatment, *Mtb* infection, Ag38kDa recombinant antibody treatment, rifampicin treatment, and combination treatment between Ag38kDa recombinant protein antibodies Rifampicin using Flowcytometry. The results of measuring the cellular immune response are shown in Table 1 and Fig 2.

**Table 1:** The results of ANOVA analysis of cellular immunity in PBMC cultures ^a^ Sign. is considered significant if p-value < 0.05

Treatment	Th1		Th2		Th17		Treg		Macrophage	
	Mean±SD	Sig.	Mean±SD	Sig.	Mean±SD	Sig.	Mean±SD	Sig.	Mean±SD	Sig.
**Control**	96.42 ± 0.80	**0.00^a^**	88.84 ± 2.61	**0,337**	51.72 ± 2.09	**0.00^a^**	95.05 ± 2.62	**0.001^a^**	47.39 ± 3.87	**0.00^a^**
**Mtb**	88.79 ± 2.30		87.90 ± 2.30		45.13 ± 4.24		90.36 ± 2.48		39.38 ± 1.54	
**Mtb+AB**	55.68 ± 4.65		85.49 ± 4.60		32.03 ± 2.44		82.15 ± 7.10		35.90 ± 1.12	
**Mtb+RF**	74.84 ± 3.11		86.84 ± 3.64		41.14 ± 2.24		90.31 ± 2.55		41.68 ± 1.00	
**Mtb+AB&RF**	98.91 ± 0.43		85.28 ± 2.44		45.34 ± 3.69		95.08 ± 1.49		57.21 ± 0.94	

**Figure 3:**
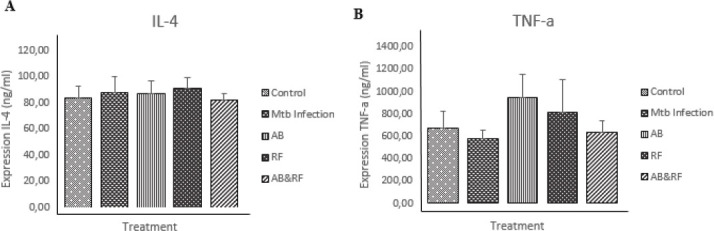
Humoral immunity from the treated groups with Ag38kDa recombinant protein antibody (AB), Rifampicin (RF), and the combination of recombinant antibody with Rifampicin (AB&RF) compared to control and *Mtb* infection.

We also examined IL-4 concentrations in Th2 humoral response and TNF-α as a Th1 humoral response. The levels of humoral immunity were examined using the ELISA method. Figure 3 shows no significant change in IL-4 levels, but TNF-α levels increased in combination treatment compared to *Mtb* infection.

**Table 2:** The results of ANOVA analysis of humoral immunity in PBMC cultures.

Treatment	IL-4		TNF-a	
	Mean±SD	Sig.	Mean±SD	Sig.
**Control**	83.89 ± 8.89	**0,448**	674.15 ± 146.49	**0.063**
**Mtb**	87.81 ± 11.61		581.18 ± 75.51	
**Mtb+AB**	86.95 ± 9.47		944.01 ± 210.38	
**Mtb+RF**	90.80 ± 7.96		815.33 ± 290.20	
**Mtb+AB&RF**	81.72 ± 5.15		629.96 104.50	

^a^ Sign. is considered significant if p-value < 0.05. There is no significant differences among the treatment As the sign is absent throughout the table.

**Figure 4:**
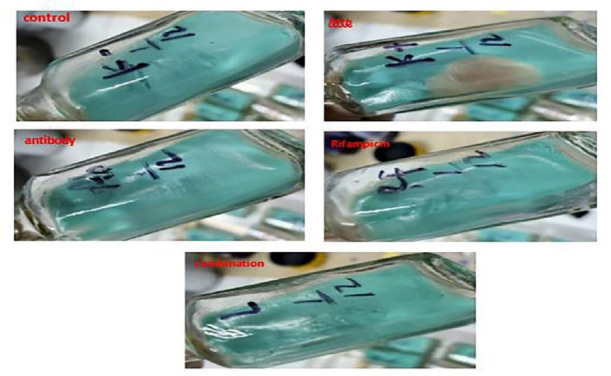
The growth of Mtb on LJ medium. Sample were taken from all groups: without the administration of bacteria ; *Mtb* infection group; Ag38kDa recombinant protein antibody group; the rifampicin group; group given a combination of recombinant antibodies with Rifampicin.

Culture results on LJ media showed that the rifampicin treatment or the combination of Rifampicin and Ag38kDa recombinant protein antibody did not show any colony growth as in control (Fig 4).

## Discussion

Based on the morphological picture on the first day after infection bacteria on macrophages, lymphocytes, and ECM, granulomas formation in our method is faster than the methods from other studies. Microscopic images show differences between treatments. Fig 1A is a control showing a complete picture of cells consisting of macrophages and lymphocytes, while Fig 1B is a treatment with *Mtb* infection showing cells that form aggregates (granulomas). Fig 1C is the treatment with Ag38kDa recombinant protein antibody showing the cells look intact without any aggregates. Fig 1D is the treatment with Rifampicin showing broken and irregular cells because Rifampicin is given after the aggregate is formed. While Fig 1E is the treatment with the combination of recombinant antibodies and Rifampicin, some cells are still intact, and some cells are broken. The results show that the administration of recombinant antibodies, Rifampicin, or a combination of Rifampicin and antibodies can inhibit bacteria through the opsonization of antibodies. While, Rifampicin can penetrate granulomas to kill bacteria. These mechanisms can eliminate *Mtb* bacteria so that the bacteria die and do not form granulomas as a sign of tuberculosis. Resolution of the granulomatous response may occur when the antigen is eliminated or a change in the local immune response and inflammation is resolved (Roman and Perez, 2019).

The results of the flow cytometry analysis showed that the percentage of macrophage cells using the CD14 marker in the combination treatment increased significantly compared to the control group, *Mtb* infection, recombinant antibody, and Rifampin. Allegedly due to the mechanism of action of antibodies that bind to bacteria in the infection area, these antibodies bind to FcyR on macrophages to advantageously modulate macrophage activation (Jacobs *et al.*, 2016). This increased macrophage activation also increases the activation of CD4+ T cells, particularly Th1 and Th17 cells (Murray and Wynn, 2011; Arnold, Gordon, Barker and Wilson, 2015). Our results showed that in the combination group, proinflammatory immune cells were increased more than those treated with Rifampin alone or antibodies alone. It is suspected that the presence of antibodies that synergize with Rifampicin causes the process of bacterial elimination to be faster and more efficient than the rifampin group or antibodies alone.

Th1, Th17, and Treg cells play a role in the immune response to *Mtb* infection and granuloma formation. The results show that Th1 cells contribute to TB protection by secreting IFN-γ and activating antimicrobials in macrophages. In addition, Th1 cells mediate the IFN-γ response in starting *Mtb* killing in vitro and contributing to *Mtb* growth restriction *in vivo* (Lyadova & Panteleev, 2015). In a previous study, Oxidized Carbon Nanosphere (OCN) formulation with two kinds of *Mtb* proteins, Ag85B and HspX, increased Th1, IFN-γ. while, levels of the Th2 cytokine, IL-5, were significantly reduced (**Sawutdeechaikul *et al.*, 2019**). In contrast, Th17 cells play a role in protection against intracellular bacteria (Acosta-Rodriguez *et al.*, 2007; Pitta *et al.*, 2009). Several studies have shown that Th17 cells are the primary producers of the IL-17 cytokine. If IL-17 synergizes with other proinflammatory cytokines, it will play a role in immunity to *Mtb* (Khader *et al.*, 2007; Scriba *et al.*, 2008; Paidipally *et al.*, 2009), such as TNF-α (Zambrano-Zaragoza *et al.*, 2014). Treg cells play a role in regulating the immune response by inhibiting the action of effector T cells (Shalev, Schmelzle, Robson and Levy, 2011). The decrease of our results study in the percentage of Th2 cells was not statistically significant. The effect of the IL-4 cytokine as the primary Th2 secreted cytokine can interfere with antimicrobial function by decreasing TNF-α mediated apoptosis in infected cells, reducing iNOS activity, and increasing iron availability for intracellular *Mtb*. Both inhibit Th2 cell components (Rook, 2007). The analysis of the humoral immune system showed a significant increase in the concentration of TNF-α, and we found no significant changes in the levels of IL-4. This result is supported by several studies showing that TB patients who experience an increase in cytokines produced by Th1 cells have a low disease severity. Meanwhile, patients who experience an increase in IL-4 cytokines have a high severity of disease (Dlugovitzky *et al.*, 1997; Dlugovitzky *et al.*, 1999).

The Lowenstein-Jensen (LJ) culture showed growth only in the group given bacterial infection, while there was no growth in the other groups. This finding indicates that Ag38kDa recombinant protein antibody, Rifampin, and the combination can eradicate bacteria in the treatment group. No growth occurs on LJ culture media.

In summary, combining recombinant antibodies with Rifampicin is interesting in developing new anti-*Mtb* treatments by giving passive vaccine candidates to accelerate the recovery of tuberculosis patients. Our study provides exciting results that *ex vivo* administration of the recombinant Ag38kDa antibody can inhibit infection with the pathogen *Mtb*, thereby reducing the cellular immune response.

### Ethical Approval

The Ethics Committee of the Faculty Medicine, Universitas Brawijaya Malang Indonesia, approved this study via an approval letter No 363/ EC/KEPK/10/2019.

### Conflict of Interest

The authors declare that they have no conflict of interest associated with this study.

**Abbreviations:** Peripheral Blood, ononuclear Cell, LJ: Lowenstein-Jensen, PBMC: Peripheral Blood, ononuclear Cell, ECM: Extracellular Matrix, RPMI: roswell park memorial institute, MOI: Multiplicity of Infection, *Mtb*: *Mycobacterium tuberculosis*,
